# Performance of diagnostic tools for acute cholangitis in patients with suspected biliary obstruction

**DOI:** 10.1002/jhbp.1096

**Published:** 2021-12-21

**Authors:** Christina J. Sperna Weiland, Celine B. E. Busch, Abha Bhalla, Marco J. Bruno, Paul Fockens, Jeanin E. van Hooft, Alexander C. Poen, Hester C. Timmerhuis, Devica S. Umans, Niels G. Venneman, Robert C. Verdonk, Joost P. H. Drenth, Thomas R. de Wijkerslooth, Erwin J. M. van Geenen, the Dutch Pancreatitis Study Group

**Affiliations:** ^1^ Department of Gastroenterology and Hepatology Radboudumc Nijmegen The Netherlands; ^2^ Department of Research and Development St. Antonius Hospital Nieuwegein The Netherlands; ^3^ Department of Gastroenterology and Hepatology Hagaziekenhuis The Hague The Netherlands; ^4^ Department of Gastroenterology and Hepatology Erasmus Medical Centre Rotterdam the Netherlands; ^5^ Department of Gastroenterology and Hepatology Amsterdam Gastroenterology Endocrinology Metabolism Amsterdam UMC Amsterdam the Netherlands; ^6^ Department of Gastroenterology and Hepatology Leiden University medical Centre Leiden The Netherlands; ^7^ Department of Gastroenterology and Hepatology Isala Clinics Zwolle The Netherlands; ^8^ Department of Surgery St. Antonius Hospital Nieuwegein The Netherlands; ^9^ Department of Gastroenterology and Hepatology Medisch Spectrum Twente Enschede The Netherlands; ^10^ Department of Gastroenterology and Hepatology St. Antonius Hospital Nieuwegein The Netherlands; ^11^ Department of Gastroenterology and Hepatology Netherlands Cancer Institute ‐ Antoni van Leeuwenhoek hospital Amsterdam The Netherlands

**Keywords:** biliary tract diseases, cholangiopancreatographies, cholangitis, diagnoses and examinations, endoscopic retrograde, validation study

## Abstract

**Background:**

Acute cholangitis is an infection requiring endoscopic retrograde cholangiopancreatography (ERCP) and antibiotics. Several diagnostic tools help to diagnose cholangitis. Because diagnostic performance of these tools has not been studied and might therefore impose unnecessary ERCPs, we aimed to evaluate this.

**Methods:**

We established a nationwide prospective cohort of patients with suspected biliary obstruction who underwent an ERCP. We assessed the diagnostic performance of Tokyo Guidelines (TG18), Dutch Pancreatitis Study Group (DPSG) criteria, and Charcot triad relative to real‐world cholangitis as the reference standard.

**Results:**

127 (16%) of 794 patients were diagnosed with real‐world cholangitis. Using the TG18, DPSG, and Charcot triad, 345 (44%), 55 (7%), and 66 (8%) patients were defined as having cholangitis, respectively. Sensitivity for TG18 was 82% (95% CI 74‐88) and specificity 60% (95% CI 56‐63). The sensitivity for DPSG and Charcot was 42% (95% CI 33‐51) and 46% (95% CI 38‐56), specificity was 99.7% (95% CI 99‐100) and 99% (95% CI 98‐100), respectively.

**Conclusions:**

TG18 criteria incorrectly diagnoses four out of ten patients with real‐world cholangitis, while DPSG and Charcot criteria failed to diagnose more than half of patients. As the cholangitis diagnosis has many consequences for treatment, there is a need for more accurate diagnostic tools or work‐up towards ERCP.

## Graphical abstract

Sperna Weiland and colleagues studied the diagnostic performance of three different tools for diagnosing acute cholangitis. Although the tools can help rule out (Tokyo Guidelines 2018) or confirm (Charcot triad and Dutch Pancreatitis Study Group criteria) acute cholangitis, there is a need for more accurate diagnostic tools or work‐up towards ERCP.

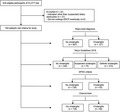

## 1 INTRODUCTION

Acute or ascending cholangitis is a bacterial infection of the biliary tract superimposed upon bile duct obstruction.^1^, ^2^ The most common cause is biliary obstruction as a result of common bile duct (CBD) stones.^2^ Acute cholangitis is a serious condition with a mortality of up to 50% when left untreated.^3^ Initial treatment comprises antibiotics and subsequent adequate biliary drainage, preferably by performing an endoscopic retrograde cholangiopancreatography (ERCP).^4^ Recent meta‐analyses reported a relation between timing of the ERCP and mortality rates, suggesting the earlier the better.^5^, ^6^ With adequate treatment a mortality of <2% can be achieved.^6^, ^7^ Therefore, diagnosing an acute cholangitis early and accurately, using diagnostic criteria with a high sensitivity and specificity is of pivotal importance.

Traditionally, acute cholangitis is diagnosed according to the Charcot triad.^8^ This relies on clinical signs: abdominal pain in the right upper quadrant, fever, and jaundice. The presence of the Charcot triad strongly suggests the presence of acute cholangitis (sensitivity 93%). Due to its low sensitivity (36%), its usefulness as diagnostic tool for acute cholangitis is limited.^9^ In 2007, the Tokyo Guideline (TG07) was issued as a novel tool for the diagnosis and severity grading for acute cholangitis.^1^, ^10^, ^11^, ^12^ Validation of the diagnostic criteria in real‐world practice showed that TG07 lacked sensitivity to identify life‐threatening cases.^13^, ^14^ The criteria for diagnosis were amended in the Tokyo Guideline 2013 (TG13). Sensitivity improved from 83% (TG07) to 92% (TG13) but did not raise specificity. A high specificity is crucial to not overtreat patients by performing an ERCP too easily which can result in unnecessary complications. The Tokyo Guidelines 2018 (TG18) used the similar definitions as in TG13.^15^ The Dutch Pancreatitis Study Group (DPSG) created diagnostic criteria for acute cholangitis in the presence of acute biliary pancreatitis for the development of the APEC trial, which have never been validated in a cohort composed of patients with or without acute biliary pancreatitis.^16^

Currently, the American and European Society of Gastrointestinal Endoscopy (ASGE and ESGE) guideline on CBD stones recommends performing an ERCP to obtain biliary drainage in case of acute cholangitis determined by TG18.^17^ Because an ERCP comes with concomitant risks, it is necessary to have a strong indication,^18^, ^19^, ^20^, ^21^ however a validation of the TG18 and DPSG criteria is still lacking. In addition, available literature shows us that there is still no evidence‐based and a sufficiently accurate guideline of clinical importance.

We aimed to evaluate the diagnostic performance of TG18, DPSG criteria, and the Charcot triad for diagnosing acute cholangitis in patients with (suspected) biliary obstruction, using real‐world diagnosis as the reference standard. Additionally, we assessed the performance of the individual criteria used in the guideline.

## 2 MATERIALS AND METHODS

### 2.1 Study design

In this study, we did a retrospective analysis of prospective data from a multicenter, parallel‐group, open‐label, superiority randomized controlled trial performed in the Netherlands.^22^ In brief, this trial has evaluated whether aggressive periprocedural hydration with lactated Ringer's solution in addition to the standard prophylactic treatment with rectal non‐steroidal anti‐inflammatory drugs reduces the risk of post‐ERCP pancreatitis in 826 patients at moderate‐ to high‐risk undergoing an ERCP. The institutional research board (Medical Research Ethics Committees United) gave permission to execute the study (NL52341.100.15, W21.171). Performance characteristics for the diagnostic tools for acute cholangitis were reported according to the Standards for the Reporting of Diagnostic Accuracy Studies (STARD) statement.^23^

### 2.2 Study population

We included patients aged 18–85 years, from 21 Dutch hospitals, who underwent an ERCP between June 2015 and June 2019 for the indication of cholangitis and/or a (suspected) biliary obstruction. Written informed consent was obtained from all patients. (Suspected) biliary obstruction was defined as: benign stricture of the bile duct, biliary tract adenoma, cholangiocarcinoma, choledocholithiasis, IgG4‐cholangiopathy, metastatic cancer, pancreatic adenocarcinoma, papillary stenosis, primary sclerosing cholangitis, and ampulla adenoma or adenocarcinoma. Patients who eventually did not undergo ERCP or had ongoing acute pancreatitis were excluded.

### 2.3 Data collection

Data were prospectively collected using a standardized data collection form and verified by the study coordinator through patient chart review. These data included: age at the time of the ERCP, sex, body mass index (BMI), the indication of ERCP, and the underlying disease established during ERCP. For this analysis, additional data were abstracted from patient charts for each eligible subject: body temperature (in °C) and/or chills, jaundice (total bilirubin >3 mg/dL^24^ or as described in physical examination), abdominal pain in the right upper quadrant, latest biochemical tests and abdominal imaging before ERCP, and antibiotic treatment indicated for cholangitis before ERCP or after ERCP when purulent bile was visualized.

### 2.4 Study endpoints and definitions

The primary study outcome was the diagnostic performance of various diagnostic tools (TG18, DPSG criteria, and Charcot triad) for acute cholangitis and real‐world diagnosis as reference standard. For details regarding these tools see Supplementary Appendix. Since there is no gold standard for the diagnosis of acute cholangitis, the clinical diagnoses made by the treating clinicians were considered to be the real‐world diagnoses. The treating clinicians took the following factors into consideration: present illness, physical examinations, laboratory data, diagnostic imaging, and clinical courses. For diagnosis of real‐world acute cholangitis a patient should at least have liver test abnormalities and the requirement of antibiotic treatment according to treating clinicians. This definition is in line with previous performed validation studies.^13^, ^14^, ^25^, ^26^ In addition, two investigators (CJSW and CBEB) independently reviewed and evaluated all of these cases. In case of discrepancies between the two investigators, these cases were discussed.

Secondary endpoint included the performance of the individual criteria used in the guideline. In addition, we evaluated in which proportion of the patients with acute cholangitis, who underwent ERCP for (suspected) choledocholithiasis, a biliary obstruction was found during ERCP.

### 2.5 Statistical analysis

Data of continuous variables are shown as mean with standard deviation (SD) when normally distributed and shown as median with interquartile range (IQR) when not normally distributed. We evaluated whether thr treatment center appeared as a confounding factor by using a binary logistic regression model. The diagnostic performance of individual risk stratification of the three diagnostic tools and per individual variable was estimated in terms of sensitivity, specificity, positive predictive value (PPV), negative predictive value (NPV), positive and negative likelihood ratios (±LR), and diagnostic accuracy. For all variables, 95% confidence intervals (CIs) were calculated. The Clopper–Pearson method was used to calculate CIs for sensitivity, specificity, and accuracy. The log method for the likelihood ratio and the standard logit was used for the predictive values. The overall performance scores for the three diagnostic tools was evaluated by estimating the corresponding area under the receiver operator characteristics (ROC) curve. Separate analyses were performed for TG18 definite acute cholangitis and TG18 suspected and definite acute cholangitis combined. We reported the risk ratio (RR) for associations between the individual risk stratification criteria and the final diagnosis of acute cholangitis. This analysis was not possible for the criteria abnormal liver function because nearly all patients met this criterion. A two‐sided *P*‐value <.05 was considered statistically significant. Statistical analyses were performed using SPSS version 26 (IBM Corp.,) and MedCalc version 19.1.2 (MedCalc Software bv).

## 3 RESULTS

### 3.1 Patient selection

**FIGURE 1:**
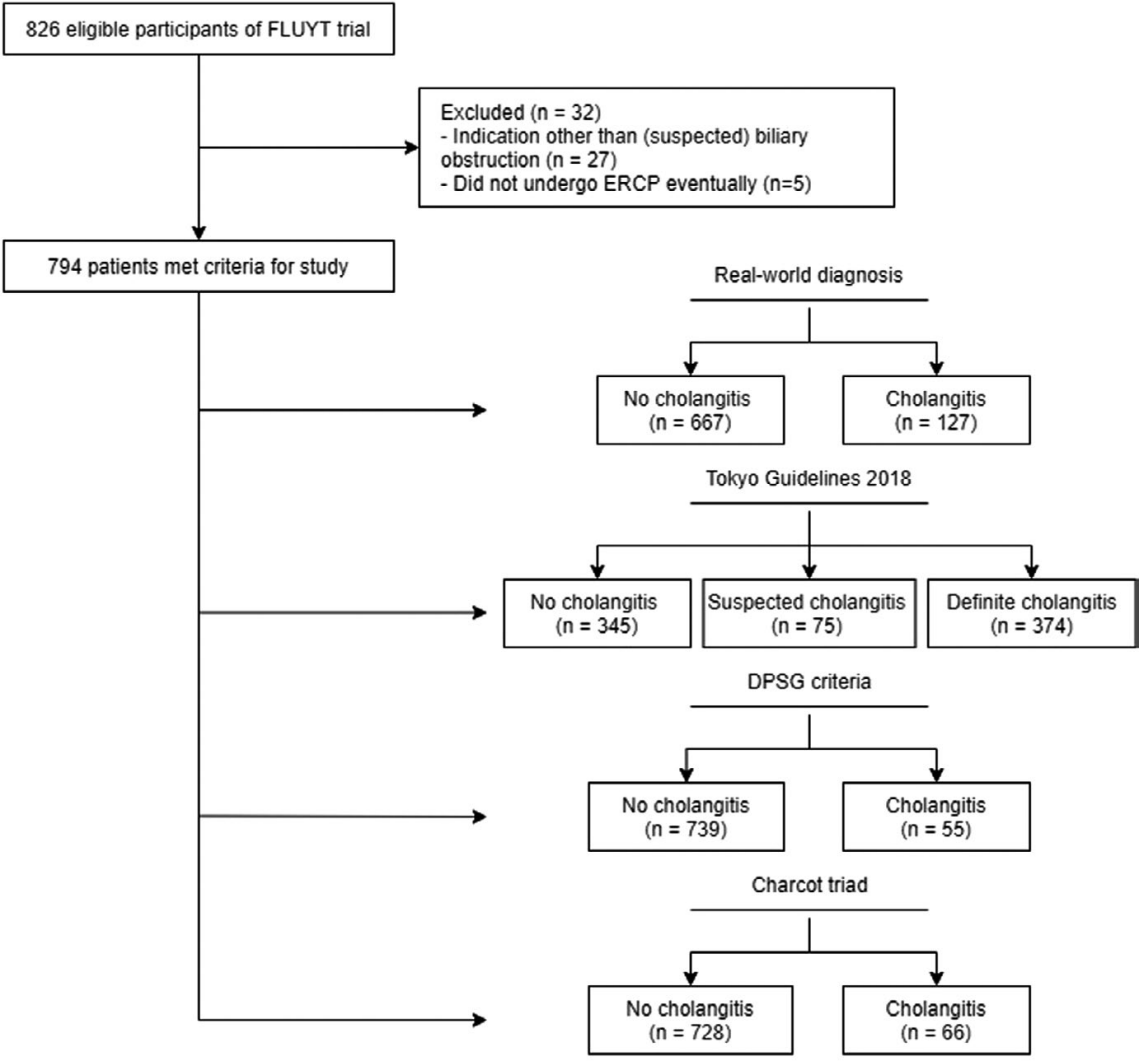
Patient selection and diagnosis per diagnostic tool. ERCP, endoscopic retrograde cholangiopancreatography. DPSG, Dutch Pancreatitis Study Group

Out of the 826 patients enrolled in the FLUYT trial, 27 patients were excluded because the ERCP was performed for reasons other than a (suspected) biliary obstruction. We excluded five patients because ERCP was ultimately not performed. Consequently, 794 patients were included in this analysis (Figure 1).

### 3.2 Patient characteristics

Patient characteristics and diagnostic outcomes are displayed in Table 1. At baseline, the median age of patients was 60 years (IQR 46.8‐71.7), and 469 (59%) patients were female. The main ERCP indication was suspected choledocholithiasis with or without cholangitis (752 patients (95%)). In the majority of these patients (74%) gallstones were visualized during the ERCP procedure. Among the 139 patients with cholangitis as ERCP indication, the majority of 136 patients (98%) had concomitant choledocholithiasis as indication, two patients (1%) had both cholangitis and cholangiocarcinoma as indication and the remaining patient (1%) had solely cholangitis as indication. Treatment center did not appear to be a confounding factor for the clinical diagnosis of acute cholangitis (*P* =.47).

**TABLE 1:** Patient characteristics and diagnostic outcomes of study cohort

	Total (n = 794)
Age (years), median (IQR)	59.6 (46.8‐71.7)
Female sex	469 (59%)
BMI (kg/m^2^), median (IQR)	26.8 (23.9‐30.3)
Length of hospital stay (days), median (IQR)	2 (1‐2)
Indication of ERCP	
Choledocholithiasis	752 (95%)
Cholangitis	127 (16%)
Benign stricture bile duct	4 (<1%)
IgG4‐cholangiopathy	1 (<1%)
Primary sclerosing cholangitis	4 (<1%)
Biliary tract adenoma	1 (<1%)
Cholangiocarcinoma	14 (2%)
Metastatic cancer	12 (2%)
Pancreatic adenocarcinoma	1 (<1%)
Papillary stenosis	2 (<1%)
Ampullary adenoma	4 (<1%)
Ampullary adenocarcinoma	2 (<1%)
Laboratory tests	
White blood cell count <4 or >10 × 1000/µL	177 (26%)
C‐reactive protein >1.0 mg/dL	377 (57%)
Aspartate aminotransferase >1.5 ULN	155 (23%)
Cholangitis according to at least one criteria	
No	453 (57%)
Yes	341 (43%)
Gallstones on ERCP indicated for choledocholithiasis	
No	173 (23%)
Yes	553 (74%)

Note

Number of missing values: BMI, 9 (1%); Length of hospital stay, 2 (<1%); Gallstones on ERCP, 26 (4%); White blood cell count, 125 (16%); C‐reactive protein, 133 (17%); Aspartate aminotransferase, 110 (14%).

Abbreviations: BMI, Body Mass Index; ERCP, endoscopic retrograde cholangiopancreatography; IQR, interquartile range; ULN, upper limit of normal.

### 3.3 Primary and secondary endpoints

All essential individual criteria per diagnostic tool were available, therefore, we did not make assumptions for stratifying patients according to the diagnostic tools (see Table S1). In total, 127 patients (16%) were diagnosed with a real‐world diagnosis of acute cholangitis. The TG18 reported definite cholangitis in 374 cases (47%). In addition, 75 patients (9%) met the criteria for suspected cholangitis. According to the DPSG criteria, 55 patients (7%) were diagnosed as having acute cholangitis. At last, 66 patients (8%) complied with the definition of cholangitis according to the Charcot triad.

In patients with the indication of choledocholithiasis and classified as having acute cholangitis according to real‐world diagnoses, the ERCP showed a biliary obstruction in 77% of the patients (see Table S2). The proportion of patients with a biliary obstruction observed during ERCP was comparable for patients with choledocholithiasis indication but classified according to the TG18 (80%), DPSG criteria (80%), or Charcot triad (83%).

### 3.4 Diagnostic performance

The accuracy of the different tools in diagnosing acute cholangitis is summarized in Table 2. The sensitivity for the TG18 definite or the combination of TG18 definite and suspected was high (82% (95% CI: 74‐88) and 98% (95% CI: 94‐100), respectively). Nevertheless, the specificity was low (60% (95% CI: 56‐63) and 51% (95% CI: 48‐55)). For the DPSG criteria and Charcot triad we found comparable diagnostic performances with a sensitivity of 42% (95% CI: 33‐51) and 47% (95% CI: 38‐56), and specificity of 99.7% (95% CI: 99‐100) and 99% (95% CI: 98‐100), respectively. The PPV for the TG18 (28%) was substantially lower compared to the DPSG criteria (96%) or Charcot triad (89%). The NPV for all the diagnostic tools was above 90%. The accuracy of the Charcot triad and DPSG criteria for diagnosing acute cholangitis was the best (91% and 90%, respectively).

**TABLE 2:** Diagnostic performance (with 95% CI) of diagnostic tools for prediction of acute cholangitis

Diagnostic Tool	Sensitivity	Specificity	PPV	NPV	LR+	LR‐	Accuracy
TG18 (susp+def)	98 (94‐100)	51 (48‐55)	28 (26‐30)	99 (98‐100)	2.03 (1.9‐2.2)	0.03 (0.01‐0.1)	59 (55‐62)
TG18 (def)	82 (74‐88)	60 (56‐63)	28 (25‐30)	95 (92‐96)	2.02 (1.8‐2.3)	0.3 (0.2‐0.4)	63 (60‐67)
DPSG	42 (33‐51)	99.7 (99‐100)	96 (87‐99)	90 (89‐91)	139 (34.4‐563.8)	0.6 (0.5‐0.7)	90 (88‐92)
Charcot triad	46 (38‐56)	99 (98‐100)	89 (80‐95)	91 (89‐92)	44 (20.7‐94.7)	0.5 (0.5‐0.6)	91 (88‐93)

Abbreviations: CI, confidence interval; def, definite; LR‐, negative likelihood ratio; LR+, positive likelihood ratio; NPV, negative predictive value; PPV, positive predictive value; susp, suspected; TG18, Tokyo Guideline 2018.

**FIGURE 2:**
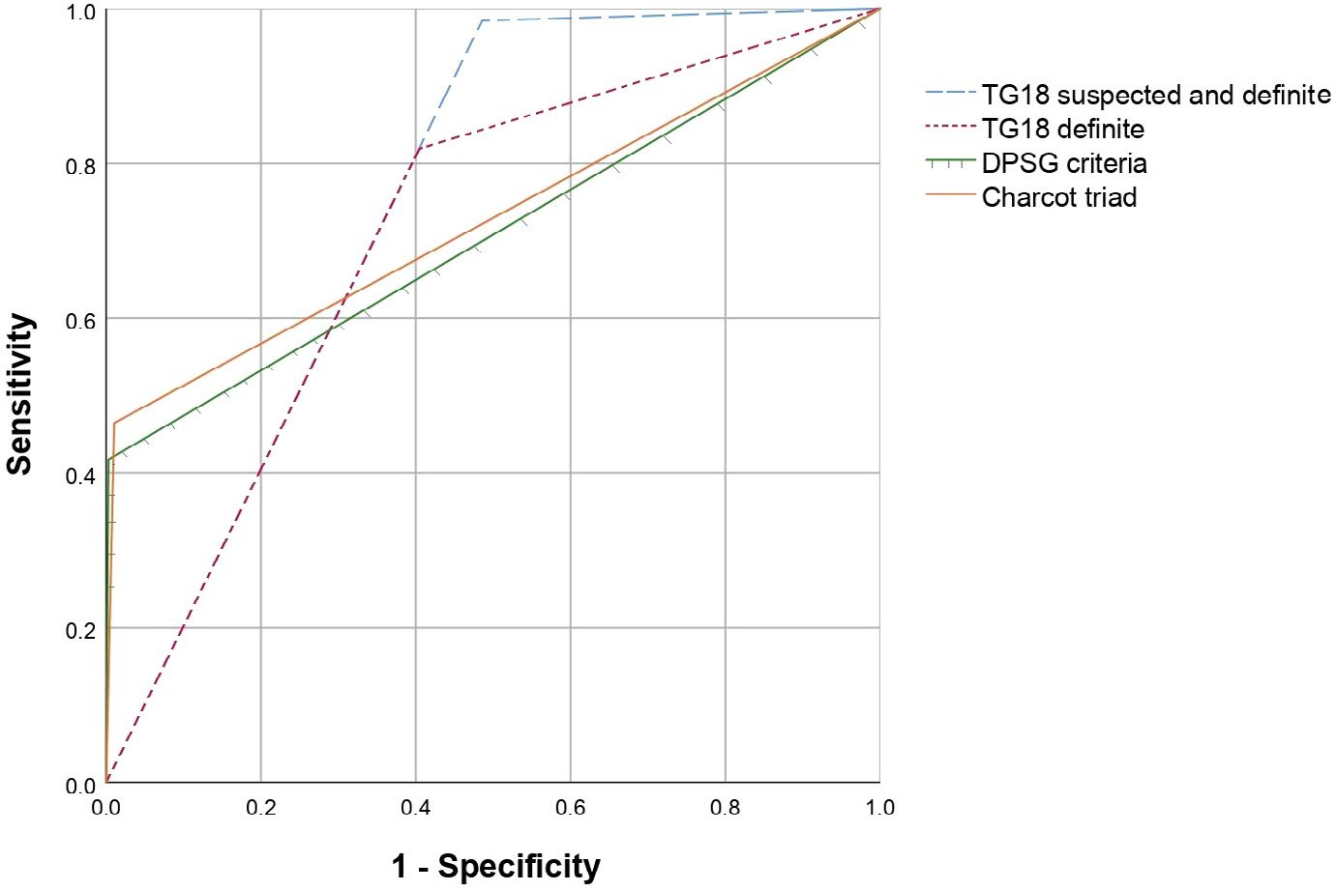
Receiver operator characteristics curves of diagnostic tools. TG, Tokyo guideline; DPSG, Dutch Pancreatitis Study Group. Area under the curves: TG18 suspected and definite diagnoses (0.75; 95% CI 0.71‐0.79), TG18 definite cholangitis (0.71; 95% CI: 0.66‐0.75), the DPSG criteria (0.71; 95% CI: 0.65‐0.77), and Charcot triad (0.73; 95% CI: 0.67‐0.79)

Figure 2 shows the ROC curves for diagnosing acute cholangitis according to the different diagnostic tools. The area under the curve was the highest for TG18 when combining suspected and definite criteria (0.75; 95% CI: 0.71‐0.79). This was followed by the Charcot triad (0.73; 95% CI: 0.67‐0.79), TG18 definite cholangitis (0.71; 95% CI: 0.66‐0.75), and the DPSG criteria (0.71; 95% CI: 0.65‐0.77).

### 3.5 Association between individual criteria and acute cholangitis

In patients with fever (> 38°C), 84% had a real‐world diagnosis of acute cholangitis. This translates into an RR of 26.8 (95% CI, 17.5‐41.0) to have cholangitis. A significant association with acute cholangitis was reported in patients with an inflammatory response, defined as leukocytes <4 or >10 ×10^9^/L or C‐reactive protein ≥10 mg/L, (RR 9.3; 95% CI, 4.1‐20.7), serum bilirubin >2 mg/dL (RR 1.5; 95% CI, 1.1‐2.1), jaundice (RR 2.4; 95% CI, 1.7‐3.5), and abdominal pain (RR 4.2; 95% CI, 2.9‐6.1). CBD dilation (RR 0.98; 95% CI, 0.7‐1.4), and obstruction of CBD on imaging (RR 0.9; 95% CI, 0.7‐1.2) were not significantly associated with cholangitis. The sensitivity and specificity of all individual criteria are on display in Table 3.

**TABLE 3:** Association between individual criteria and real‐world cholangitis (prevalence 16%)

	n/N	Cholangitis	Sensitivity (95% CI)	Specificity (95% CI)	RR (95% CI)
Body temperature >38°C	126/794	84%	83 (76‐89)	97 (95‐98)	26.8 (17.5‐41.0)
Inflammatory response^a^	448/659	26%	95 (90‐98)	38 (34‐43)	9.3 (4.2‐20.7)
Total bilirubin >2 mg/dL	418/710	21%	68 (59‐76)	43 (39‐47)	1.5 (1.1‐2.1)
CBD dilation	554/773	16%	69 (60‐77)	30 (27‐34)	0.98 (0.7‐1.4)
Obstruction of CBD on imaging	501/773	15%	61 (52‐69)	36 (33‐40)	0.90 (0.65‐1.25)
Jaundice	399/786	23%	71 (63‐79)	53 (49‐57)	2.42 (1.69‐3.48)
Abdominal pain	329/792	29%	75 (66‐82)	65 (61‐68)	4.18 (2.87‐6.08)

Abbreviations: CBD, common bile duct; CI, confidence interval; n, number of cases; N, total number of cases; RR, risk ratio.

^a^ Inflammatory response: Leukocytes <4 or >10 × 10^9^/L or C‐reactive protein ≥1 mg/dL.

## 4 DISCUSSION

We found that four out of ten patients would be incorrectly diagnosed with acute cholangitis by applying the TG18. The Charcot triad or DPSG criteria performed worse, and more than half of patients with cholangitis would be misdiagnosed. Nevertheless, all diagnostic tools are able to rule out the diagnosis of cholangitis with a high probability.

This study has several strengths. First, all included patients were participating in a large nationwide prospective multicenter randomized trial.^22^ This is the first diagnostic accuracy study evaluating TG in a Western population. Previously performed diagnostic accuracy studies on the TG were all performed in Asia and, therefore, might not be generalizable to the Western population.^27^ Furthermore, we included patients with all types of biliary obstructions, not only patients with suspected choledocholithiasis. This gives a better representation of the total population at risk for acute cholangitis.

Some limitations of this analysis should be acknowledged. First, there is no known infallible gold standard for diagnosing acute cholangitis. We used real‐world diagnosis of cholangitis as the reference standard which might be influenced by interpretation. However, this is in line with previous performed validation studies and we addressed this issue partly by including objective criteria (start of antibiotic treatment for acute cholangitis) as a condition of our reference standard.^13^, ^14^, ^25^, ^26^ In addition, our study population consists of patients all undergoing an ERCP. Even though the ERCP is the preferred manner for achieving biliary drainage, not all patients with a cholangitis will eventually be treated with an ERCP. This supposedly is a minority of patients, but these patients were not portrayed in our cohort. Secondly, we might underestimate patients scored as having acute cholangitis according to the DPSG and Charcot criteria. This could be due to the early start of antibiotic treatment in the emergency department, which potentially suppressed the inflammatory response and body temperature during hospital admission.

The TG18 shows an acceptable sensitivity (82%) and a moderate specificity (60%) in diagnosing definite cholangitis. Recently, a retrospective study performed in Japan and Taiwan, compared TG13 and TG07 in a cohort of patients with real‐world acute cholangitis.^25^ Here the TG13 diagnostic criteria possessed a superior diagnostic ability to diagnose acute cholangitis (90% (TG13) vs. 79% (TG07); (*P* <.0001)). Another study yielded similar results as our study and achieved a sensitivity of 84% (TG13) and 51% (Charcot triad).^28^ This study enrolled cases with an acute cholangitis on the basis of purulent bile visualized during ERCP. The threshold for cholangitis is perhaps too high as purulent bile is not universally present in acute cholangitis cases.

In clinical practice, acute cholangitis is a clear indication to perform an ERCP according to the ESGE guideline.^17^ The handling of the TG18 as a diagnostic tool will possibly result in unnecessary ERCPs in 20% of the patients with concomitant suspicion of CBD stones (see Table S2). Thereby, disregarding the ERCP‐related complications that can occur, such as post‐ERCP pancreatitis (3.5%‐9.7%), bleeding (0.3%‐9.6%), perforation (0.08%‐0.6%), and anesthesia‐related adverse events (0.02%).^18^, ^19^, ^20^, ^21^ Acute cholangitis has a broad clinical presentation and is, therefore, difficult to capture in a diagnostic guideline. Additional imaging with endoscopic ultrasound (EUS) or magnetic resonance cholangiopancreatography (MRCP) might be a less invasive alternative for initial evaluation and to assess the need for ERCP in these patients. As is shown through available literature and current practice, there is no sufficiently accurate guideline of clinical importance for acute cholangitis. We reported a significant overtreatment with antibiotics and/or ERCP when applying TG18, while Charcot triad and DPSG criteria lead to untreated cholangitis patients. Nevertheless, all the diagnostic tools are useful to rule out acute cholangitis due to their high NPVs. The high PPVs of the DPSG, and to a lesser extent of the Charcot triad, allow clinicians to confirm with considerable certainty the diagnosis of acute cholangitis.

Future research to improve and validate the existing guidelines should be executed in a prospective design, in which microbiological analysis and severity grading of cholangitis should be taken into account. By focusing on developing a new diagnostic tool, it should be taken into account that we found five of the individual diagnostic criteria (fever, total bilirubin >2 mg/dL, inflammatory response, jaundice, and abdominal pain) to be statistically significantly associated with acute cholangitis. Most of these criteria are in line with the recently suggested BILE criteria (Biliary abnormalities or intervention, Inflammatory marker elevation, Liver tests abnormalities, and Exclusion of cholecystitis and acute pancreatitis) to identify patients with high probability of cholangitis.^29^ Additionally, it would be meaningful to focus on an indicator to optimize timing of biliary drainage in subgroups of patients with cholangitis.

In conclusion, the international guidelines recommend to perform directly prompt ERCP in patients with a clinical ascending cholangitis. However, the use of TG18, DPSG, or Charcot triad as a conclusive diagnostic tool will lead to a high number of incorrectly diagnosed (TG18) or missed acute cholangitis patients (DPSG/Charcot). It can help clinicians to rule out (TG18) or confirm acute cholangitis (DPSG and Charcot). Nevertheless, we advise clinicians to consider to perform additional imaging, by EUS or MRCP, before ERCP in patients with acute cholangitis.

## CONFLICT OF INTEREST

Authors declare no conflict of interests for this article.

## AUTHOR CONTRIBUTIONS

All authors meet all four criteria for authorship: (a) Substantial contributions to the conception or design of the work, or acquisition, analysis or interpretation of data for the work; (b) Drafting the work or revising it critically for important intellectual content; (c) Final approval of the version to be published; (d) Agreement to be accountable for all aspects of the work in ensuring that questions related to the accuracy or integrity of any part of the work are appropriately investigated and resolved.

## Supporting information

Supplementary Material

## Data Availability

Request for data can be made to the corresponding author and will be discussed during a meeting of the Dutch Pancreatitis Study Group. Individual participant data that underlie the results reported in this article, after de‐identification, will be shared after approval of the Dutch Pancreatitis Study Group.
