# Supplementary material for: Performance of diagnostic tools for acute cholangitis in patients with suspected biliary obstruction
Source: J Hepatobiliary Pancreat Sci. 2021 Dec 21;29(4):479–86. doi: 10.1002/jhbp.1096 (PMC9306734; doi:10.1002/jhbp.1096)
Supplement: Supplementary file 1 — Supplementary Material [file JHBP-29-479-s001.docx]

**Supplementary Appendix**

This appendix has been provided by the authors to give readers additional information about their work.

**Performance of diagnostic tools for acute cholangitis in patients with suspected biliary obstruction**

Christina J. Sperna Weiland, Celine B.E. Busch, Abha Bhalla, Marco J. Bruno, Paul Fockens, Jeanin E. van Hooft, Alexander C. Poen, Hester C. Timmerhuis, Devica S. Umans, Niels G. Venneman, Robert C. Verdonk, Joost P.H. Drenth, Thomas R. de Wijkerslooth, Erwin J.M. van Geenen, on behalf of the Dutch Pancreatitis Study Group.

**Contents**

[Tokyo Guideline 2007 for acute cholangitis 3](#_Toc71800814)

[Tokyo Guideline 2013 for acute cholangitis 3](#_Toc71800815)

[Tokyo Guideline 2018 for acute cholangitis 3](#_Toc71800816)

[Charcot triad 4](#_Toc71800816)

[DSPG criteria for cholangitis 4](#_Toc71800817)

[Table S1. Diagnostic tool versus clinic-based diagnosis of acute cholangitis 4](#_Toc71800818)

[Table S2. Biliary obstruction during ERCP for the indication of choledocholithiasis in patients with cholangitis according to a diagnostic tool 4](#_Toc71800819)

| **Tokyo Guideline 2007 for acute cholangitis** |
| --- |
| A. Clinical context and clinical manifestations  *1. History of biliary disease*  *2. Fever (>38°C) and/or shaking chills*  *3. Jaundice*  *4. Abdominal pain (RUQ or upper abdominal)*  B. Laboratory data  *1. Evidence of inflammatory response (abnormal WBC count, increased serum CRP level, and other changes indicating inflammation)*  *2. Abnormal liver function tests (increased serum ALP, γ-GTP, AST, and ALT levels)*  C. Imaging findings  *1. Biliary dilatation, or evidence of the etiology on imaging (stricture, stone, stent etc.)* |
| Suspected diagnosis: Two or more items in A  Two or more items in A + both items in B and item C |
| WBC=white blood cell, CRP=C-reactive protein, ALP=Alkaline phosphatase, *γ*GTP=r-glutamyltransferase, AST=aspartate aminotransferase, ALT=alanine aminotransferase, RUQ=right upper quadrant, ULN=upper limit of normal. |

| **Tokyo Guideline 2013 for acute cholangitis** |
| --- |
| A. Systemic inflammation  *1. Fever (>38°C) and/or shaking chills*  *2. Laboratory data: evidence of inflammatory response (WBC <4 or >10 x1,000/µL OR CRP ≥1mgl/dL)*  B. Cholestasis  *1. Jaundice (Bilirubin ≧2 (mg/dL)*  *2. Laboratory data: abnormal liver function tests (ALP, γGTP, AST, ALT >1.5 ULN)*  C. Imaging  *1. Biliary dilatation*  *2. Evidence of the etiology on imaging (stricture, stone, stent etc.)* |
| Suspected diagnosis: one item in A + one item in either B or C  Definite diagnosis: one item in A, one item in B and one item in C |
| WBC=white blood cell, CRP=C-reactive protein, ALP=Alkaline phosphatase, *γ*GTP=r-glutamyltransferase, AST=aspartate aminotransferase, ALT=alanine aminotransferase, ULN=upper limit of normal. |

| **Tokyo Guideline 2018 for acute cholangitis** |
| --- |
| A. Systemic inflammation  *1. Fever (>38°C) and/or shaking chills*  *2. Laboratory data: evidence of inflammatory response (WBC <4 or >10 x1,000/µL OR CRP ≥1mgl/dL)*  B. Cholestasis  *1. Jaundice (Bilirubin ≧2 (mg/dL)*  *2. Laboratory data: abnormal liver function tests (ALP, γGTP, AST, ALT >1.5 ULN)*  C. Imaging  *1. Biliary dilatation*  *2. Evidence of the etiology on imaging (stricture, stone, stent etc.)* |
| Suspected diagnosis: one item in A + one item in either B or C  Definite diagnosis: one item in A, one item in B and one item in C |
| WBC=white blood cell, CRP=C-reactive protein, ALP=Alkaline phosphatase, *γ*GTP=r-glutamyltransferase, AST=aspartate aminotransferase, ALT=alanine aminotransferase, ULN=upper limit of normal. |

| **Charcot Triad** |
| --- |
| Fever (>38°C) and/or shaking chills  Jaundice  Abdominal pain (right upper quadrant or upper abdominal) |

| **DSPG criteria for cholangitis** |
| --- |
| Highest in-hospital body temperature in previous 24 hours: ≥ 38.5°C with chills, without an  obvious other cause (e.g., cystitis, pneumonia, thrombophlebitis, etc), or 39ºC without chills,  without an obvious cause for fever, **and either:**  1) Choledocholithiasis on abdominal US, CT, EUS or MRI, **OR**  in the absence of gallstones and/or sludge  2) A dilated common bile duct on imaging defined as >8mm in patients ≤75 years or >10mm in patients >75 years **OR**  3) Progressive cholestasis for at least two consecutive days and a bilirubin >2.3 mg/dL (40 μmol/L) |
| DPSG=Dutch Pancreatitis Study Group, CT=computed tomotograhy, US=Ultrasound, EUS=Endoscopic ultrasonography, MRI=Magnetic resonance imaging. |

| **Table S1. Diagnostic tool versus clinic-based diagnosis of acute cholangitis** | | | |
| --- | --- | --- | --- |
|  | **Clinic-based cholangitis** | |  |
|  | *No* | *Yes* | *Total* |
| According to TG18: suspected and definite | | | |
| No | 343 | 2 | 345 |
| Yes | 324 | 125 | 449 |
| According to TG18: only definite | | | |
| No | 397 | 23 | 420 |
| Yes | 270 | 104 | 374 |
| According to DPSG criteria | | | |
| No | 665 | 74 | 739 |
| Yes | 2 | 53 | 55 |
| According to Charcot triad | | | |
| No | 660 | 68 | 728 |
| Yes | 7 | 59 | 66 |
| TG=Tokyo guideline, DPSG=Dutch Pancreatitis Study Group | | | |

| **Table S2.** **Biliary obstruction during ERCP for the indication of choledocholithiasis in patients with cholangitis according to a diagnostic tool** | | | | | |
| --- | --- | --- | --- | --- | --- |
|  | **Clinical based diagnoses** | **TG18 definite** | **TG18 suspected and definite** | **DPSG criteria** | **Charcot triad** |
| Biliary obstruction* | 96 (77%) | 283 (80%) | 325 (75%) | 44 (80%) | 54 (83%) |
| No biliary obstruction | 26 (21%) | 70 (20%) | 101 (23%) | 10 (18%) | 11 (17%) |
| Missing | 2 (2%) | 3 (<1%) | 5 (1%) | 1 (2%) | 0 (0%) |
| Total patients | 124 | 356 | 431 | 55 | 65 |
| TG = Tokyo guideline, DPSG = Dutch Pancreatitis Study Group  * definded as: gallstones, benign stricture bile duct, IgG4cholangiopathy, primary sclerosing cholangitis, biliary tract adenoma, cholangiocarcinoma, metastatic cancer, auto-immune pancreatitis, pancreatic adenocarcinoma, papillary stenosis, ampullary adenoma, ampullary adenocarcinoma. | | | | | |
